# Huge left atrial myxoma: about 2 cases resected in the Democratic Republic of Congo

**DOI:** 10.1186/s13019-023-02286-2

**Published:** 2023-04-20

**Authors:** Alphonse Ndonga N’sungu Nzomvuama, Cédric Nyombo Mutuale, Roger Minga Kongo, Irène Nsolani Mbanzulu, Joseph Makunza Nsiala

**Affiliations:** 1 Department of Thoracic and Cardiovascular Surgery, Kinshasa University Hospital, University of Kinshasa, BP 834, Kinshasa, Democratic Republic of the Congo; 2 Department of Cardiovascular Surgery, Ngaliema Clinic, Kinshasa, Democratic Republic of the Congo; 3 Hassan II University Hospital, Fès, Morocco; 4 Department of Histopathology, Kinshasa General Hospital, Kinshasa, Democratic Republic of the Congo; 5 Department of Anesthesiology, Kinshasa University Hospital, University of Kinshasa, Kinshasa, Democratic Republic of the Congo

**Keywords:** DR Congo, Left atrial myxoma, Surgery

## Abstract

**Background:**

Myxoma is the most common cardiac tumor, found in 75–80% of cases in the left atrium. It can grow quietly and therefore reach a large size before being symptomatic. Poor availability of echocardiography also contributes to delayed diagnosis. In Sub-Saharan African countries, myxoma diagnosis can be missed for many patients. Myxoma resection surgery, although technically simple, is not always possible, because of the lack of cardiac surgery development. The aim of this report is to describe the first two consecutive resection cases of huge left-atrial myxoma performed in Kinshasa, Democratic Republic of Congo (DRC) and to discuss the specificities of this surgery in this low-resource context.

**Case presentation:**

Two patients, 54 and 48 years old, were diagnosed with giant myxoma of the left atrium in the management of progressive dyspnea The first patient's transthoracic echocardiography revealed a pedunculated atrial mass (37 × 48 mm) on the interatrial septum, passing through the mitral valve. For the second patient, the mass (64 × 26 mm) was attached to the roof of the left atrium and protruded into the mitral valve, with significant mitro-tricuspid regurgitation The first patient underwent a simple resection of the myxoma. For the second patient, it was associated to a mitro-tricuspid annuloplasty. The postoperative course was simple for the first patient, but the second patient developed a biventricular failure requiring vasoactive drugs. Both patients were discharged alive from the hospital on postoperative days 10 and 12, respectively. They are regularly followed up and are doing well 2 years after surgery.

**Discussion and conclusion:**

Surgical resection is the only effective treatment of myxoma. Our first results are encouraging The poor availability of the echocardiography is a challenge in the diagnosis of myxoma. The development of cardiac surgery in DRC and ongoing country-level efforts to address diagnostic challenges for these often silent tumors will allow us to expect more resections to be performed locally and larger series published.

## Background

Myxoma is the most common cardiac tumor, found in 75–80% of cases in the left atrium. Prevalence is estimated between 0.001% and 0.3% [1]. Myxomas can quietly grow to a large size and then become symptomatic. Surgery prevents from severe complications, such as embolic accidents or the risk of sudden death [2]. Surgical outcomes are generally simple and recurrences rare [3].

In Sub-Saharan African countries, this technically simple surgery may not always be possible, because of the lack of cardiac surgery development. Few patients afford the opportunity to undergo surgical treatment abroad [4, 5]. For the others, there remains two alternatives: expect a providential surgery performed by a humanitarian mission or wait patiently for a certain death [4].

The aim of this report is to describe the first two consecutive resection cases of huge left-atrial myxoma performed in Kinshasa, Democratic Republic of Congo (DRC) and to discuss the specificities of this surgery in this low-resource context.

## Case 1

**Fig. 1:**
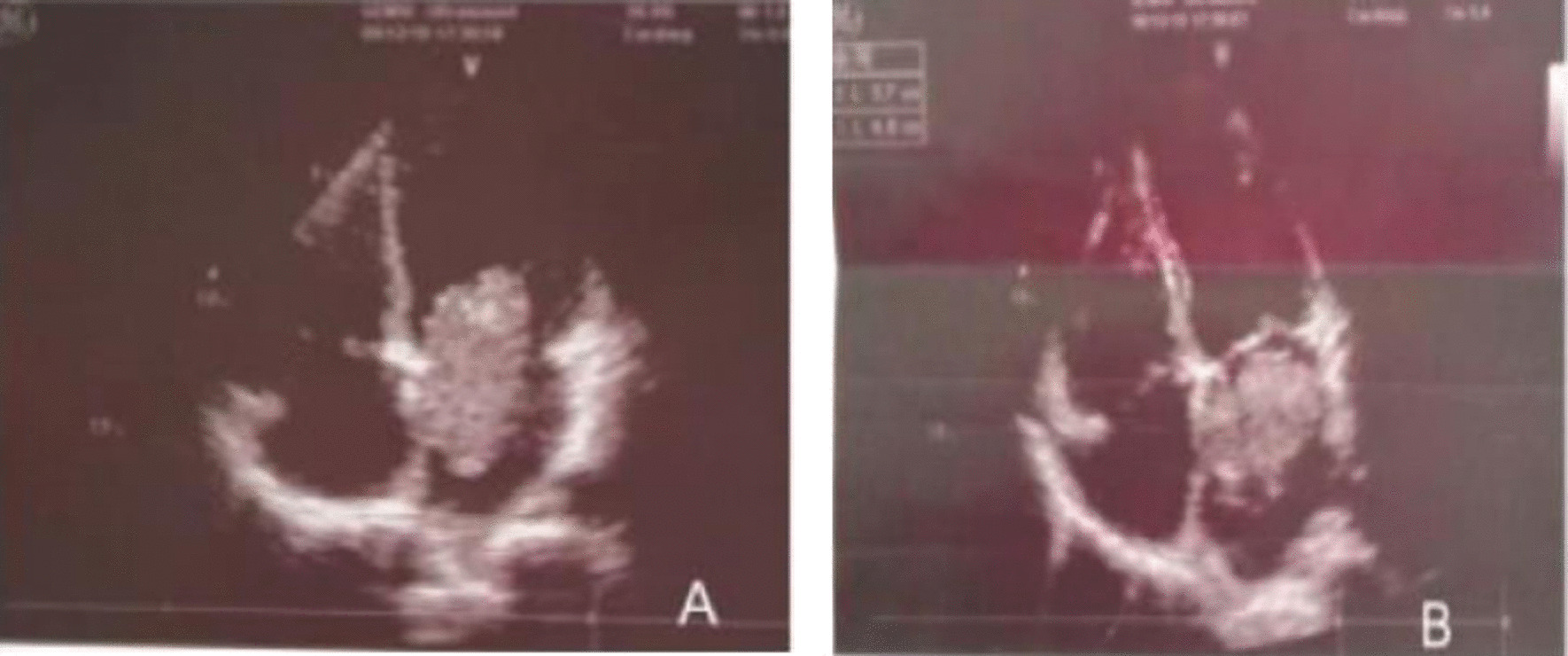
Preoperative transthoracic echocardiography (TTE) showing a pedunculated atrial heart mass (37 × 48mm) on the interatrial septum, protruding through the mitral valve (**A**) and for the case 2 (**B**), a large mass (64X26mm) attached to the roof of the left atrium

A 54-year-old man, a Jehovah's Witness, consulted for progressive dyspnea (NYHA III) and palpitations. He had a history of atrial fibrillation (AF) and had been on Acenocoumarol (Sintrom™). A systolic murmur was audible. Preoperative transthoracic echocardiography (TTE) revealed a pedunculated atrial mass (37 × 48 mm) on the interatrial septum. It passed through the mitral valve and was associated with minimal mitral regurgitation (Fig. 1A). The left ventricular ejection function (LVEF) was preserved (60%).

## Case 2

A 48-year-old man complained of progressive dyspnea (NYHA III), palpitations and productive cough. A systolic murmur (3/4) was heard on cardiac auscultation. A large mass (64 × 26 mm) attached to the roof of the left atrium was observed at the TTE. It was protruding into the mitral valve (Fig. 1B). There was significant mitro-tricuspid regurgitation with pulmonary hypertension (PAPs = 56 mmHg) and a preserved LVEF (55%).

## Surgery and outcomes

**Fig. 2:**
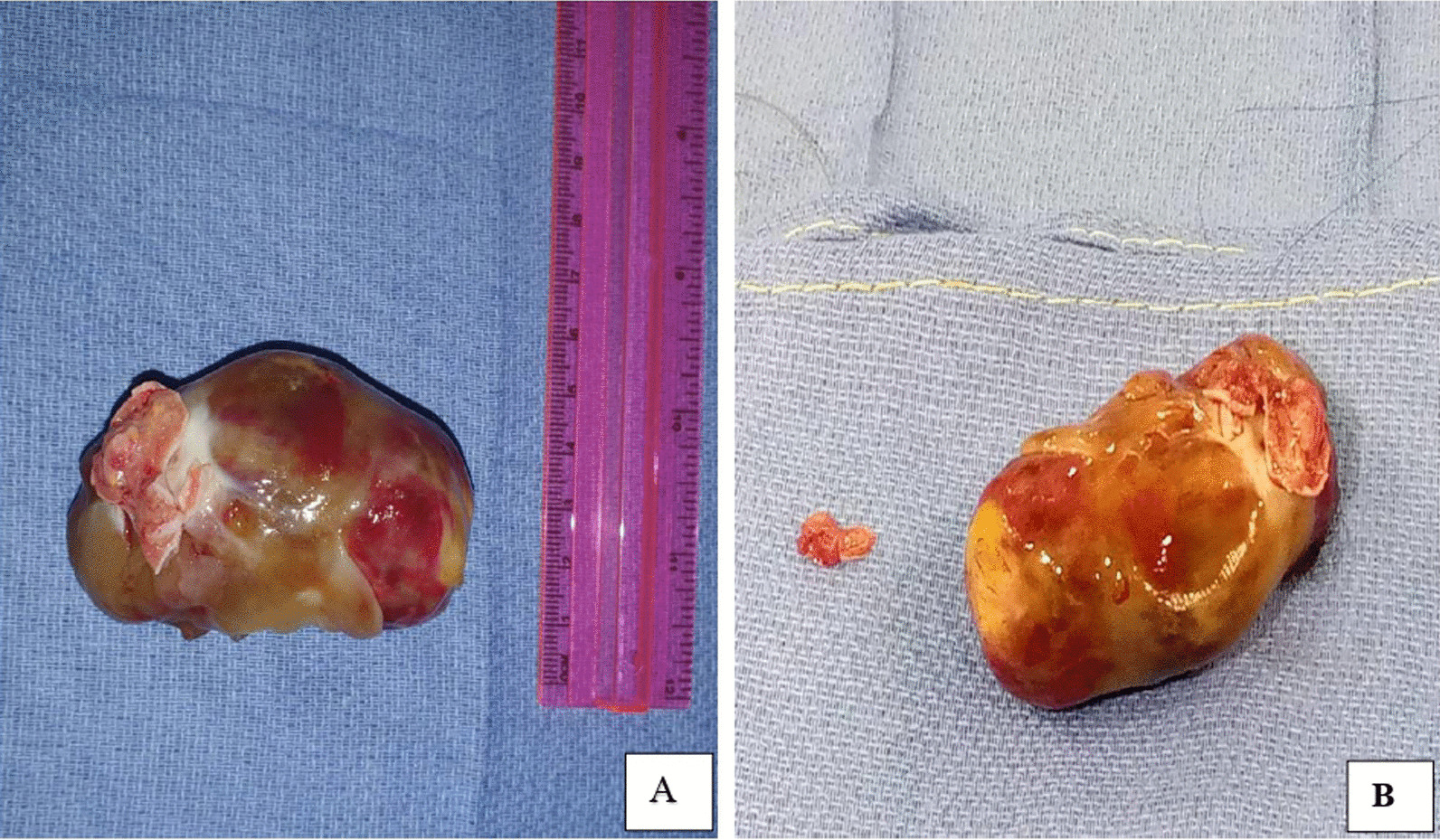
Myxomas removed. Case 1 (**A**) and case 2 (**B**)

Sternotomy was the approach for both patients. Access to the left atrium was achieved by a right atriotomy and the atrial septum incision. Tumors were resected, removing their implantation base (Fig. 2A and B). For the second patient, annuloplasty rings corrected mitral and tricuspid regurgitations. Peroperative use of the blood recovery and autotransfusion system (Cell Saver 5+™) avoided the first patient's transfusion, while respecting his religious convictions.

The postoperative course was uneventful for the first patient. At day 1 after surgery TTE confirmed the absence of any residual mass and no mitral regurgitation. He was discharged from hospital on day 10.

For the second patient, the postoperative course was marked by a bi-ventricular failure and the occurrence of the AF. Cardiac failure required vasoactive drugs for 3 days and AF was resolved with amiodarone. The postoperative TTE, at postoperative day 1, revealed no residual mass but just a trivial mitral valve leak. The patient was discharged at day 12.

**Fig. 3:**
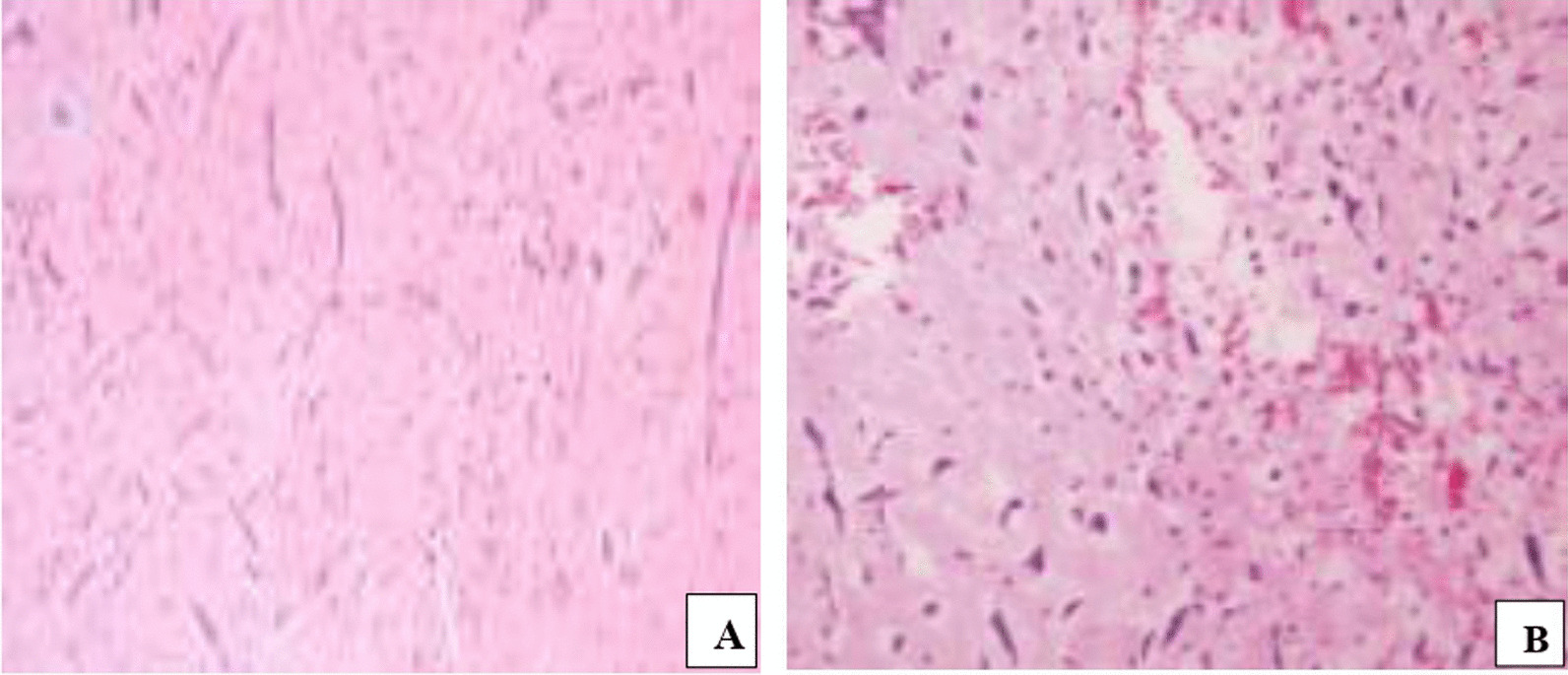
Hematoxylin and eosin staining, magnification ×40 (**A**) and ×100 (**B**): an abundant myxomatous matrix containing polygonal and spindled cells with eosinophilic myxiod cytoplasm. Nuclei are elongated, rounded, or oval and its staining characteristic varied from pale to intensely hyperchromatic. Scattered inflammatory cells were also present. No nuclear atypia nor mitotic activity were seen (Hematoxylin and eosin stain; original magnification 10, for all three panels)

Histological examination established that in both cases it was myxoma (Fig. 3). The two patients are followed up regularly. Two years following surgery, they are both doing well and no tumor recurrence has been observed.

## Discussion and conclusion

This report describes the first two myxoma resections performed in the DRC. A Congolese team carried out surgeries. Since 2016, there is in Kinshasa a cardiac surgery center where the surgeries we have described were performed in 2019. Due to lack of resources, operations depend essentially on international humanitarian missions. As in the DRC, a few cardiac surgery centers have been opened in recent years in Sub-Saharan Africa, allowing patients to have surgery at home [5].

Two cases of myxoma resections may appear anecdotal. But in fact these two cases provide evidence that Congolese surgeons are able to perform cardiac surgery in their country. We can wonder about the fate of Congolese patients with myxoma over time. For most of them, myxoma generally went undiagnosed.

Early diagnosis of atrial myxoma may be missed because its evolution remains silent for a long time. Several publications report cases of incidental diagnosis. In some cases, this clinical discretion allows tumor to grow to large sizes. Clinical signs and even complications of myxoma depend on tumor size, location, and shape. A huge left atrial myxoma can also mimic the clinical signs of mitral stenosis [6, 7].

TTE is currently the standard diagnostic test in myxoma. However, TTE is not yet widely available in our country. It is mainly practiced in 4 or 5 major cities where experienced cardiologists have established practice. We can reasonably consider that myxoma diagnosis is ignored for many patients. The DRC has a currently estimated population between 84 and 95 million people. With increased availability of TTE, there is a legitimate expectation that there will be more diagnosis and resection of myxomas in the future.

Both patients in our reports were diagnosed with stage III NYHA dyspnea. TTE identified huge left atrial tumors prolapsing to the left ventricle. For the second patient, the tumor led to mitral annular dilatation, which resulted in significant Carpentier's Type 1 mitral regurgitation.

For our first patient, the preoperative mitral regurgitation was no longer seen at postoperative control. Indeed, after removal of the myxoma, the mitral leaflets had recovered a satisfactory coaptation. Mitral regurgitation associated with a large left atrial myxoma often resolves spontaneously after removal of the tumor. Sometimes mitral annulus dilatation by a huge myxoma may persist [8]. Such was the case for the second patient, whose mitral and tricuspid annular dilatation required correction with annuloplasty rings.

Resection of the myxoma is its only effective treatment. Access to the left atrial myxoma through the right atrium and septal incision allows a good exposure of the tumor, an easy and complete inspection of all cardiac chambers. Then, surgery consists of a complete resection of the myxoma with its implantation base.

The two patients are doing well over two years after surgery. They are followed up regularly and have resumed their regular activities and work.

We reported our first two consecutive and successful resections of huge left atrial myxomas. Our results are encouraging. The development of cardiac surgery in DRC and ongoing country-level efforts to address diagnostic challenges for these often silent tumors will allow us to expect more resections to be performed locally and larger series published.

## Data Availability

The datasets used and/or analysed during the current study are available from the corresponding author on reasonable request.
